# Retrospective Multi-Center Analysis of Canine Socket Prostheses for Partial Limbs

**DOI:** 10.3389/fvets.2019.00100

**Published:** 2019-04-05

**Authors:** Theresa M. Wendland, Bernard Seguin, Felix M. Duerr

**Affiliations:** Department of Clinical Sciences, Colorado State University, Fort Collins, CO, United States

**Keywords:** prosthesis, prosthetic, amputation, partial amputation, socket, orthotic

## Abstract

**Introduction:** Socket prostheses for treatment of distal limb pathology are becoming increasingly prevalent in veterinary medicine, however, limited objective data is available. Objectives of the present study were to retrospectively evaluate owner satisfaction, clinical outcomes, and prognostic factors associated with dogs receiving socket prostheses for partial limbs in a larger patient population.

**Materials and Methods:** Client databases of a single prosthesis provider were reviewed to identify owners whose dogs had received a prosthetic device within the last 10 years. An online survey was developed to evaluate owner-reported outcomes. The survey inquired about anatomy of the residuum, concurrent disease, prosthesis use, rehabilitation, activity, complications, and owner satisfaction. Medical records and radiographs were requested from all participants. Radiographs were used to confirm level of amputation and evaluate for osseous complications. Survey responses were analyzed by assigning author-defined numeric scores defining clinical outcome and owner satisfaction.

**Results:** One-hundred thirty-seven owners were contacted. The response rate was 50/137 (37%); 47 responses were analyzed. Forty-six of 47 owners reported positive satisfaction; 1/47 was displeased. Forty-two of 47 dogs were scored to have acceptable to full function; 5/47 had unacceptable clinical function using the author-defined scoring system. A 62% short-term complication rate and a 19% long-term complication rate were reported. Skin sores were the most common short and long-term complication. There was a significant correlation between both clinical outcome scores and owner satisfaction with days per week spent in the prosthesis. Additionally, clinical outcome scores and owner satisfaction significantly varied between dogs with different durations of prosthesis wear with a trend toward better outcomes associated with longer prosthesis wear. Radiographs were obtained for 23/47 dogs to further define level of defect. The most proximal level of defect was mid-radius for the forelimb and mid-tibia for the hind limb. There was no correlation between level of defect and either owner satisfaction or clinical outcome.

**Discussion/Conclusion:** Results of this survey suggest a high degree of owner satisfaction despite substantial complication rates. Based on preliminary data, further evaluation of socket prostheses as a limb-sparing option for treatment of distal limb pathology should be considered. Prospective clinical trials with objective outcome measures are required to draw firm conclusions.

## Introduction

The use of socket prostheses for companion animals is emerging as a more common treatment in the field of veterinary medicine (1, 2). Due to their growing prevalence and potential to change current practices in veterinary medicine, it is crucial to objectively assess the value and efficacy of socket prosthetics as a treatment option for distal limb pathology in veterinary patients.

Partial limb amputation is frequently performed in people so that a prosthesis may be used (3). Conversely, total limb amputation is considered the standard of care as a treatment for appendicular neoplasia, infection, trauma, and congenital defects affecting canine patients, even for distal limb pathology (4–10). This discrepancy in perspective and treatment approach is presumably due to bipedal vs. quadrupedal differences between people and dogs. However, recent kinetic and kinematic analyses of dogs who received total limb amputations of thoracic or pelvic limbs revealed significant alterations to locomotive biomechanics when compared to quadrupedal dogs (4, 11, 12). Such gait alterations may have deleterious effects on long-term musculoskeletal health and lead to other quality of life issues (13). These effects have been described clinically, though the long-term impact that total limb amputation has on orthopedic health has not been objectively studied. Additionally, while multiple surveys have been published in the veterinary literature indicating positive owner satisfaction with total limb amputation, owners still reported negative changes to their dog's mobility, attitude, and quality of life (6, 14–16). For example, Dickerson et al. found that 27% of owners reported a change in their dog's recreational activities, 42% reported some change in ability to maneuver stairs, 23% were unable to return to pre-amputation walking routines, 9% reported a change in their dog's attitude, and 12% reported that their dog did not return to pre-amputation quality of life (16).

It has been suggested that a prosthetic limb re-establishes quadruped gait and structure preventing development of secondary musculoskeletal disease (1). There are reports in the veterinary literature of horses, calves, and a single deer that have undergone partial amputation with socket prosthesis placement demonstrating the ability to restore quadrupedal function with such devices (17–19). To date, two retrospective studies have documented outcomes of socket prosthesis placement in dogs. One owner survey based study reported that 87.5% of patients (21/24) had the same to improved quality of life as they did prior to receipt of a prosthesis (20). Another study indicated that 83.3% of owners (10/12) reported a good to excellent quality of life following prosthesis placement (21). This may suggest that socket prosthetics could be a viable option for some canine patients. There are still many questions about prosthetic use in canine patients which have been left unanswered. While owner perceptions of quality of life and owner expectations regarding mobility have been assessed, overall owner satisfaction with prostheses as a treatment option has not yet been investigated. Another important question, which warrants further study, is the exact levels of amputation or limb defect which can be successfully treated with prosthetic devices. It is well-documented within human medicine that the level of limb defect has a strong correlation with clinical outcomes (22, 23). In the veterinary patient, suggestions of optimal limb length have been made by veterinarians and veterinary prosthetists based on clinical experience, however this has not been definitively established (20). Other prognostic factors, such as age, reason for prosthesis placement, frequency of use of the prosthesis, and whether or not rehabilitation was performed have yet to be identified.

The objectives of the present study are to retrospectively evaluate owner-perceived outcomes associated with dogs who received a socket prosthesis for a partial-limb and to identify overall owner satisfaction as well as prognostic factors for owner satisfaction and clinical outcomes. We hypothesized that overall owner satisfaction with prosthesis placement and use would be high. It was also hypothesized that distal limb defects would correlate with positive clinical outcomes and higher owner satisfaction with prosthetic devices compared to proximal limb defects.

## Materials and Methods

### Case Selection

This was a survey-based study sent to owners of dogs who had undergone a partial limb amputation, had a congenital defect, or had a partial limb due to unknown causes. Dog owners who received socket prostheses in the last 10 years from a single veterinary prosthetic manufacturer^1^ were identified by database review based on previously indicated willingness to participate in research. Identified owners were solicited via email for survey participation.

### Prosthetic Devices

**Figure 1:**
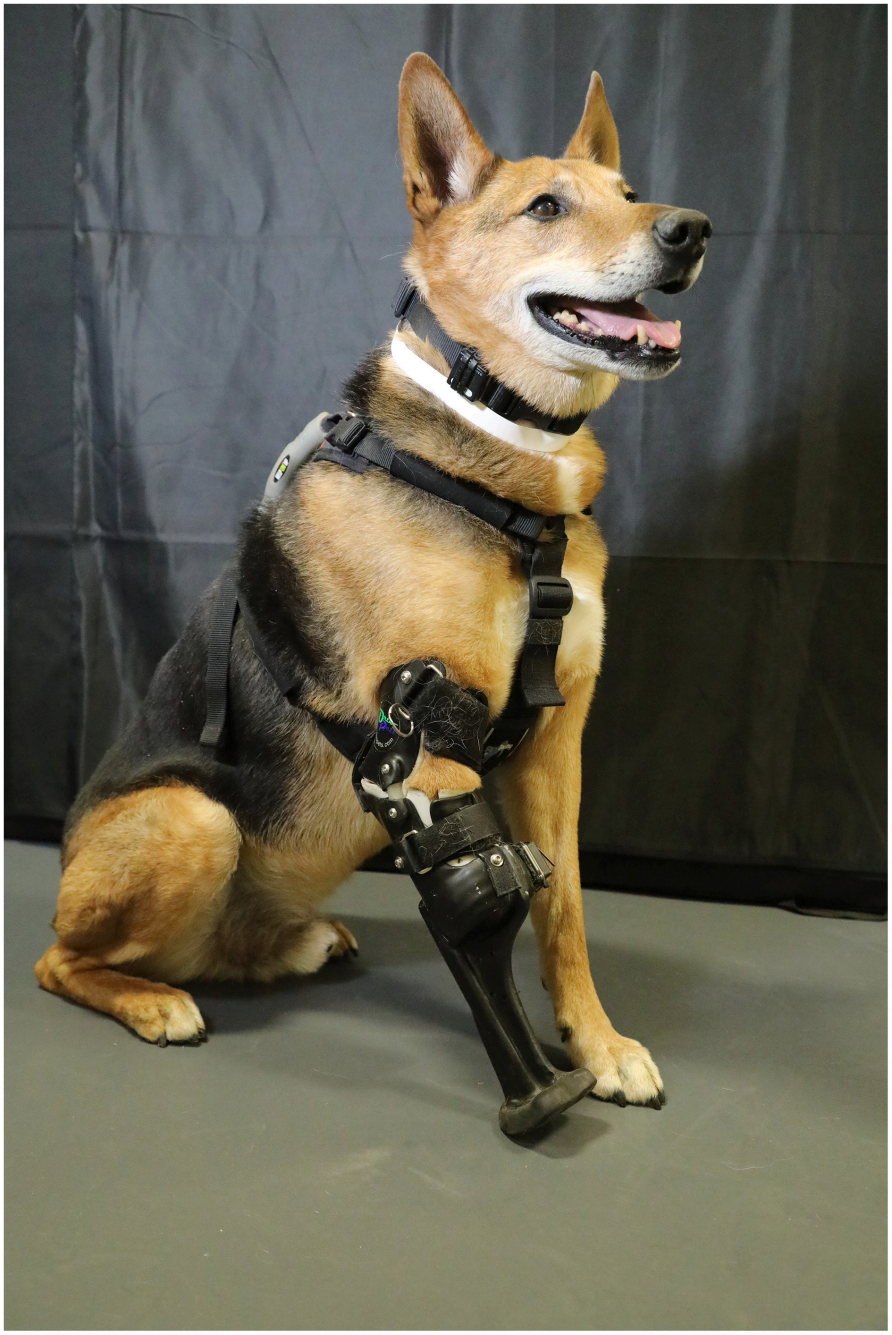
Example of a thoracic limb prosthetic device for a patient with a mid-radius amputation.

**Figure 2:**
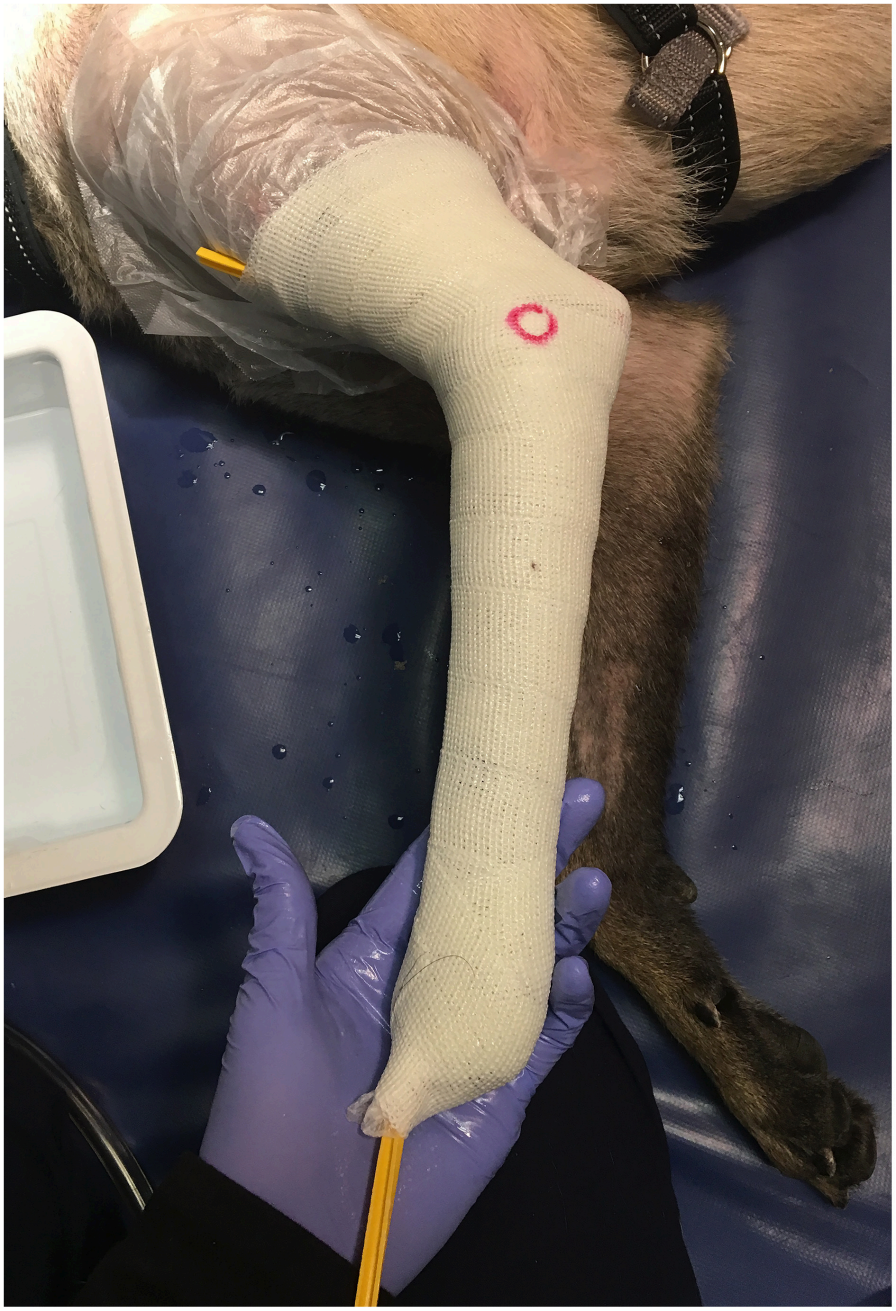
Photograph of a limb casted for a prosthetic device creating the mold used to manufacture the prosthetic.

All prostheses were custom manufactured by a single veterinary prosthetic manufacturer (Figure 1). The device was created based on a mold of the patient's residuum created out of fiberglass cast tape by the referring veterinarian (Figure 2). Initial prosthesis fitting, recommendations for prosthesis use, and aftercare associated with the device were all provided by the referring veterinarian. Complications were addressed by the veterinarian with support from the manufacturer, and any necessary revisions to the device were made by the veterinarian or by sending the device back to the manufacturer.

### Survey and Medical Records

An online survey was developed to evaluate owner-reported outcomes and satisfaction associated with socket prosthesis use by canine patients. The survey was linked in an email and administered through an online survey tool^2^. Owners who chose to participate were required to identify their pet by name, provide personal contact information, and name a veterinarian involved in the management of the prosthesis for purposes of medical record obtainment and review. Following required identification and contact information, there were 31 required survey questions.

The survey (available in Supplemental Materials) inquired about breed, affected limb, reason for partial limb, level of amputation or defect, age at receipt of prosthesis, time between limb-loss and receipt of prosthesis, time spent in the prosthesis, patient activity prior to and following receipt of prosthesis, activities performed in the prosthesis, patient acceptance of the prosthesis, rehabilitation, complications, concurrent orthopedic and neurologic disease, and owner satisfaction. Each question had a text box for optional additional comments or clarification. There was also an optional text box included at the end of the survey for additional miscellaneous comments. For the question which inquired about level of limb defect, a diagram and detailed descriptions were provided to owners to select the level of defect.

**Figure 3:**
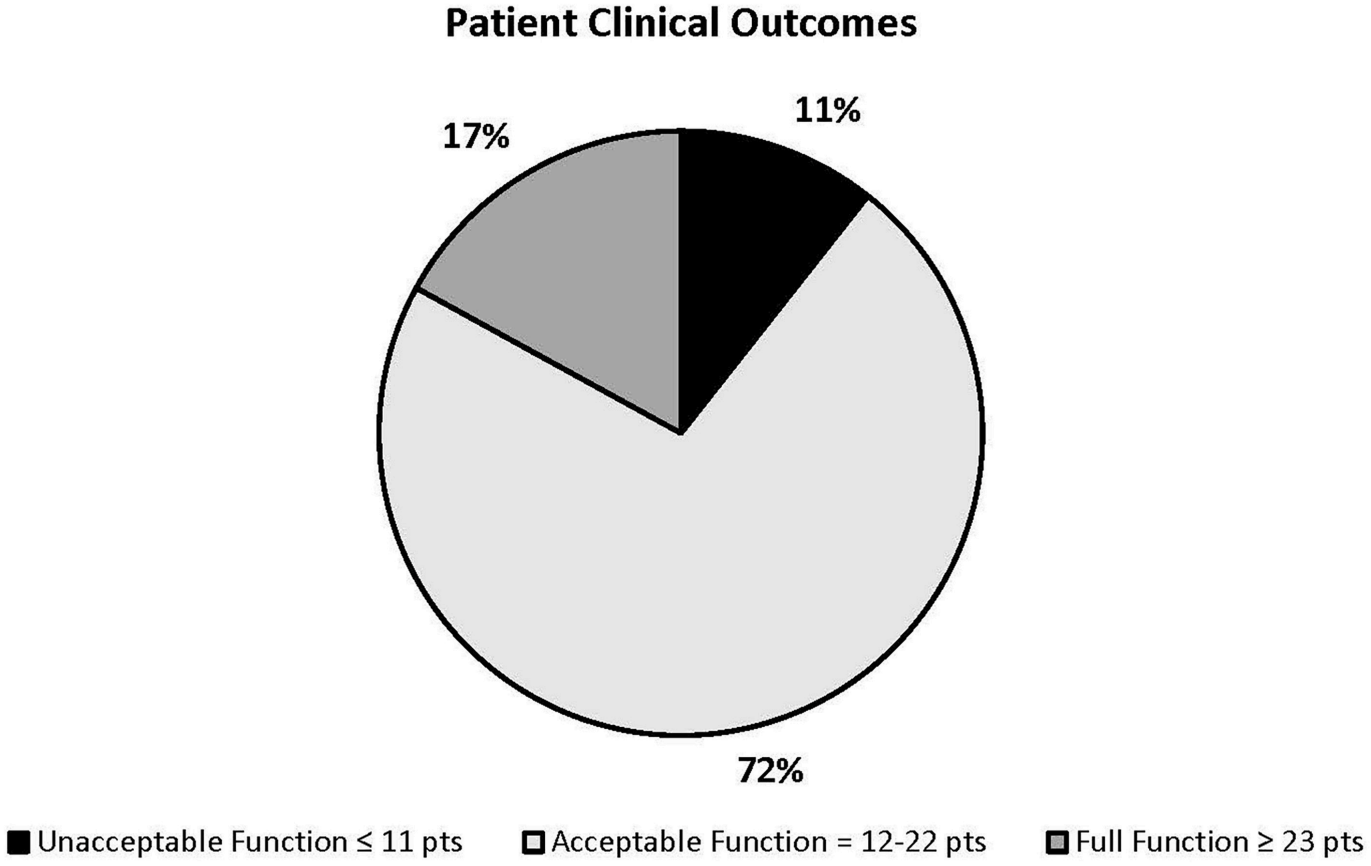
Patient clinical outcome score ranges and percentage of patients who fall into each range.

Survey responses were analyzed by assigning scores to question responses related to clinical outcome (Table 1). The scored questions included information regarding frequency and duration of prosthesis wear, walking ability in the prosthesis, activity in the prosthesis, adaptive tasks performed with the prosthesis, acceptance of prosthesis donning, and complications experienced. Each question response was assigned a numeric value. Responses were assigned positive values if the factor was considered to be associated with a favorable clinical outcome and negative values were assigned to factors considered to be associated with unfavorable clinical outcomes. Question outcomes were numerically weighted based on the clinical implications and relevance as determined by the authors. For example, each short term complication was assigned a value of negative one while each long-term complication was assigned a value of negative two. Sums were tallied for each patient. A scale was created from the scores obtained based on factors determined by previous reports in the veterinary literature and by clinical experience (6, 16, 24). Scale cut-off points were determined arbitrarily prior to data analysis by comparing hypothetical numeric values to the clinical picture of a patient that would fall into that range. A previously outlined clinical outcome scoring system was utilized as a guideline for outcome definitions: Full function was defined as restoration to, or maintenance of, full intended level and duration of activities and performance from pre-injury or pre-disease status. Acceptable function was defined as restoration to, or maintenance of, intended activities and performance from pre-injury or pre-disease status that is limited in level or duration. Unacceptable function was defined as all other outcomes (25). Outcomes were numerically defined as full function (≥23 points), acceptable function (12–22 points), and unacceptable function (≤ 11 points) with a possible score range of −13 to 34 (Figure 3).

**Table 1:** All questions that were included in the author-defined clinical outcome scoring and the points assigned to each of the possible answers provided.

**CLINICAL OUTCOME SCORING**	
**Scored question**	**Point assignments**
How many days per week is the prosthesis worn on average?	+1 point for each day per week the device is worn
Approximately how many hours per day does your dog wear the prosthesis (on days it is worn)?	Less than 1 = 0 points; 1–3 = 1point; 3–6 = 2 points; 6–9 = 3 points; 9–12 = 4 points; 12–15 = 5 points; 15–18 = 6 points
For what purpose is the prosthesis used? (You may choose more than one answer)	+1 point for each purpose selected/listed
How well does your dog walk with the prosthesis?	Never = 0 points; holds up most of the time but places some steps = 2 points; holds up for some steps = 3 points; Uses for almost every step = 4 points
How well has your dog adapted to using the prosthesis for other tasks? Please select specific tasks your dog does well with the prosthesis (you may choose more than one).	+1 point for each purpose selected/listed;−1 point if the option “My dog does not use the prosthesis well for any tasks” is selected
Does your dog like having the prosthesis placed?	Avoids having it placed = 0 points; Doesn't mind = 1 point; Excited = 2 points
How has your dog's activity changed since receiving the prosthesis? For dogs who had an amputation, please compare to your dog's lifestyle prior to limb loss (i.e.,: before the problem started).	Moderate to marked decrease = −2 points; Mild decrease = −1 point; Same or don't know = 0 points; Mild increase = 1 point; Moderate to marked increase = 2 points
Has your dog experienced any short-term prosthesis complications? Examples: pressure or rub sores that resolved in <8 weeks and did not reoccur, quickly resolving pain associated with the amputation/defect site, swelling or size fluctuations of the amputated limb <8 weeks after amputation, etc.	−1 point for every complication selected/listed
Has your dog experienced any long-term prosthesis complications? Examples: Pressure or rub sores that lasted more than 8 weeks or reoccurred, chronic pain associated with the amputation/defect site, swelling or size fluctuations of the amputated limb more than 8 weeks after amputation, etc.	−2 points for every complication selected/listed

Responses were also analyzed for owner satisfaction. The scored questions included owner-reported satisfaction level, whether or not they would chose this treatment option again for their dog, and whether they would recommend a prosthesis as a treatment option to another owner. Possible scores ranged from zero to six, with zero being considered dissatisfied and six being very satisfied (Table 2).

**Table 2:** All questions that were included in the author-defined owner satisfaction scoring and the points assigned to each of the possible answers provided.

**OWNER SATISFACTION SCORING**
**Scored question**	**Point assignments**
How would you describe your level of satisfaction with your dog's prosthesis?	Unhappy = 0 points; Acceptable = 1 point; Better than acceptable = 2 points; Very happy = 3 points
Based on your experience, would you choose a prosthesis for your dog again?	Yes = 1 point; No = 0 points
Knowing what you do now, how likely are you to recommend a prosthesis to another dog owner?	Unlikely = 0; Likely = 1; Very likely = 2 points

Clinical outcome and owner satisfaction were analyzed for correlations with other factors which may be prognostic indicators such as signalment, rehabilitation performed, concurrent disease, time between limb loss and receipt of prosthesis. Clinical outcomes and owner satisfaction were also compared against factors contained within each scored outcome, such as time spent in the prosthesis and complication rates. For statistical analysis, breeds were classified as x-small, medium, large, x-large breed, as designated by the American Kennel Club (26). If the breed description was unclear, the dog was classified as “other” and was analyzed as a separate category acknowledging that patient size was potentially highly variable. Each category was assigned a numeric value for purposes of statistical analysis.

When available, medical records and radiographs of survey respondents' dogs were obtained and reviewed for comparison with survey responses. When radiographs of the affected limb were not available, owners of dogs who were identified as still living were contacted to assess interest in having radiographs taken of the affected limb. Owners and their preferred veterinary clinics were contacted to arrange participation. Participating veterinary clinics were reimbursed for radiographs taken at their facility. All radiographs were used to compare owner-reported level of amputation or defect and to assess for radiographic signs of residual limb complications.

### Statistical Analysis

The data included in clinical outcome scores and owner satisfaction scores was analyzed using a non-parametric Kruskal-Wallis test to compare between data groupings. Spearman's Rho was used to evaluate correlation between the scores and other continuous variable. Continuous data were represented using means. However, if the data was not normally distributed then medians were used. A *p*-value of 0.05 was used for determining statistical significance. SAS v9.4^3^ was used for all statistical analyses.

## Results

One hundred thirty seven owners were identified by the prostheses manufacturer as having met the inclusion criteria. Emails with survey links were sent to 137 owners with a 50/137 (36.5%) survey completion rate. The survey was sent three times at 2 week intervals to owners who had not yet responded. Three survey responses were excluded for prosthesis non-use or minimal-use. These three exclusions involved a severe injury unrelated to the prosthesis leading to prosthesis non-use prior to device receipt and two cases in which the prosthesis was newly received and had not yet been or had been minimally used at the time the survey was completed. This resulted in 47 survey responses included in the statistical analysis.

**Figure 4:**
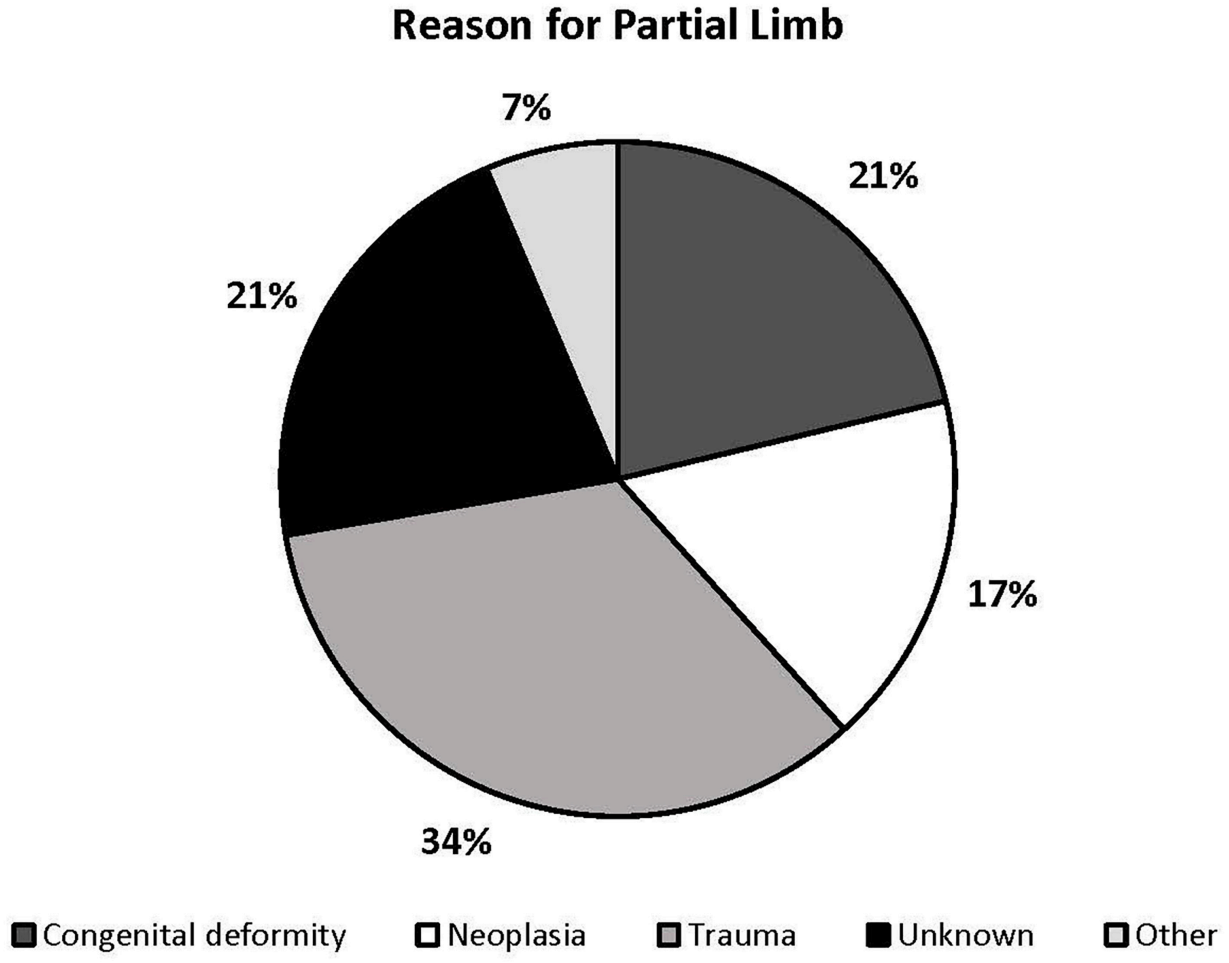
Percentage breakdown describing the reason included dogs had a partial limb.

Thirty dogs were reported to have received a partial limb amputation, 11 had a congenital defect, and 6 had an unknown history resulting in a partial limb (Figure 4). The mean age at the time of prosthesis placement was reported to be 3.8 years and the median age was 3 years. The median time range between limb loss and prosthesis placement could not be accurately described for all dogs because owners reported an unknown timeframe for 22/47 dogs. In dogs for which the timeframe was known, the median timeframe range was 2–6 months between limb loss and prosthesis placement. Twenty-seven of 47 patients underwent some form of rehabilitation therapy post-prosthesis fitting. The most common duration of rehabilitation therapy performed was 2–3 months. The remainder of descriptive data can be found in the Supplemental Material Dataset.

Twenty-five of 47 (53.2%) owners responded that they were very satisfied with the outcome, 12/47 (25.5%) reported a better than acceptable outcome, 9/47 (19.1%) reported an acceptable outcome, and 1/47 (2.1%) was displeased with the outcome. Forty-five of 47 (95.7%) reported that in hindsight, they would choose this treatment option again. Forty-two of 47 (89.3%) dogs were considered to have acceptable function or full function clinical outcomes based on author-defined clinical outcome scoring criteria, while 5/47 (10.6%) were determined to have a poor clinical outcome by the same criteria. There was a significant correlation between clinical outcome scores and owner satisfaction scores (*r*_s_ = 0.5400, *p* < 0.0001).

Dogs spent from 0 to 7 days per week in their prostheses with a median of 7 days per week. There was a significant positive correlation between clinical outcome scores and days per week spent in the prosthesis (*r*_s_ = 0.3146, *p* < 0.0313). There was an even stronger positive correlation between owner satisfaction scores and days per week spent in the prosthesis (*r*_s_ = 0.6662, *p* < 0.0001). Dogs spent 0 h per day up to 15–18 h per day in their prosthetic devices with a median range of 2–6 h per day. Clinical outcome scores varied significantly between dogs with different hour durations that the prosthesis was worn (*p* < 0.0001) with better outcomes associated with longer prosthesis wear. Similarly, owner satisfaction significantly varied between the dogs with different hour durations of prosthesis wear (*p* < 0.0258) with more positive outcomes in dogs spending the greatest number of hours in their prostheses.

Other prognostic indicators evaluated included dog size and whether or not rehabilitation was performed prior to fitting of a prosthetic device (pre-habilitation). The relationship between clinical outcome scores and dog size (*p* = 0.0898) was approaching significance, with medium breed dogs appearing to do clinically best. There was a significant difference in owner satisfaction scores and dog size (*p* = 0.026), with dogs of unspecified dog size associated with highest owner satisfaction. X-Small breed dogs and x-large breed dogs received the lowest scores in both clinical outcome and owner satisfaction. The relationship between clinical outcome scores and dogs receiving pre-rehabilitation (*p* = 0.0714) was approaching significance and there was a significant difference in owner satisfaction scores between dogs receiving pre-rehabilitation vs. those which did not receive pre-habilitation (*p* = 0.0417). Both clinical outcome scores and owner satisfaction scores were higher in dogs who did not receive pre-habilitation. Only 9/47 dogs received rehabilitation prior to receipt of their prosthesis.

Owner satisfaction scores were significantly different between categories of owner's ability to place the prosthesis (*p* = 0.0063) with ease of prosthesis placement related to owners with the highest satisfaction scores.

Level of defect, reason for partial limb, affected limb, time between limb loss and prosthesis placement, breed, age at the time of prosthesis placement, rehabilitation after prosthesis placement, or concurrent orthopedic or neurologic disease had no correlation to or significant differences with either owner satisfaction scores or clinical outcome scores. No correlations or significant differences were found between owner satisfaction scores and short- or long-term complications. Complication rates could not be statistically correlated to clinical outcome since complications were factored into this score.

There was a reported 61.7% (*n* = 29) short-term complication rate with sores being the most common complication (*n* = 21), followed by pain (*n* = 5), swelling (*n* = 2), and dermatitis (*n* = 1). A long-term complication rate of 19.1% (*n* = 9) was reported (sores *n* = 7, pain *n* = 1, and dermatitis *n* = 1).

Radiographs were obtained for 23/47 dogs to confirm and further define level of defect. All defects were found to be at the level of the mid radius/ulna or mid tibia/fibula or distal to this level. Sixteen of 23 owners (69.6%) correctly identified the exact level of limb defect, though all inaccurate reports were found to be just above or just below the joint level reported. There was no significant difference found between level of defect and either owner satisfaction scores (*p* = 0.589) or clinical outcome scores (*p* = 0.4099). A more detailed description of defect level can be found in Table 3. Only 1/23 patient radiographs of a mid-radial amputation revealed obvious radiographic signs of residual limb complication in the form of a distal residuum bursa. Veterinary records indicate that only one patient received a revision surgery to remove the bursa of the above mentioned patient.

**Table 3:** Defect levels and their respective mean Clinical and Owner Satisfaction Scores; There are greater than *n* = 47 limbs described due to multiple patients having more than one partial limb.

**Level of limb defect**	***N***	**Mean clinical outcome score**	**Standard deviation (clinical outcome)**	**Mean owner satisfaction score**	**Standard deviation (owner satisfaction)**	**Number of limbs with level confirmed radiographically**
1–Digital defects	4	15.3	7.4	5.3	1.5	4
2–At carpus/tarsus or distal to joint	32	16.4	5.8	5	1.2	19
3–Mid-radius/ulna or mid-tibia/fibula	8	17.3	4.3	5.4	1.1	1
4–Unknown	1	25	-	6	-	0

*The number of limbs and the described defect level confirmed with radiographs of the partial limb is shown in the far right column. Again, it should be noted that this column contains 24 limbs, though radiographs were only obtained for 23 subjects. One subject had multiple affected limbs for which radiographs were provided*.

## Discussion

To the authors' knowledge, this is the first multi-center study evaluating owner satisfaction with socket prosthesis use in dogs. Results of the present study suggest a high satisfaction rate among owners electing a socket prosthesis for treatment of distal limb pathology. Additionally, most owners surveyed would elect to choose this treatment option again, and would recommend the use of socket prosthetics to another dog owner. Clinical outcomes in the present study appeared slightly better than previously reported outcomes (21). However, criteria utilized to determine clinical outcome differed between studies, therefore, direct comparison of these outcome scores cannot be made.

Factors which may impact prognosis of patients receiving a socket prosthesis were also investigated. Level of amputation is of particular interest due to clinical application in making recommendations for surgical limb-sparing options. Level of limb defect has been demonstrated across multiple studies in human medicine to have a significant correlation with clinical outcomes (22, 23). In the veterinary patient, it has been recommended that socket prostheses can be considered with partial amputations as proximal as the proximal third of the radius/ulna and mid-tibia (2). These recommendations have been made based on clinical impressions of device suspension needs, limitation of planes of motion, and proprioceptive feedback to the patient (2). It has also been suggested that pelvic limbs with amputations at or below the tarsus provide benefit due to good suspension via the malleoli while a thoracic limb may be treated with a more proximal amputation due to anatomy and device suspension from the humeral condyles (20). Neither of these suggestions, however, have been supported by objective data at this time. In the recent literature, one study evaluated three dogs with mid-diaphyseal radius/ulna defects and one dog with a mid diaphyseal tibial defect (20). Specific outcomes for these dogs were not reported. Another study reported on three patients with an antebrachial defect and three patients with defects at the tarsocrural joint or proximal (21). Outcomes of 2/3 dogs with antebrachial defects were defined as good, 1/3 was poor. Outcomes of all dogs with defects at or proximal to the tarsocrural joint were defined as good; the precise level of defects was not defined for thoracic or pelvic limbs (21). Previous studies found no correlation between level of amputation and outcome measures, however, the low case numbers did not allow to draw firm conclusions (20, 21). The present study investigated a larger number of patients receiving prosthetic limbs and a combination of detailed description, diagrams, and radiographic review were used to accurately define level of amputation or defect. Despite these efforts, we were unable to establish a correlation or significant difference between level of defect and either owner satisfaction scores or clinical outcome scores. This may suggest that level of limb defect is of less importance in a quadrupedal patient than it is in a bipedal patient. On the other hand, the present study only identified eight cases with a defect at the level of the mid radius/ulna or tibia/fibula resulting in a small sample size making a Type II statistical error possible. Therefore, further investigation with a larger sample size and fewer patient variables is warranted to establish definitions of limb length and correlation to clinical outcome.

Of the possible prognostic factors investigated, time spent in the prosthetic device appeared to have the most profound association with owner satisfaction scores and clinical outcome scores with significant positive correlations. Dogs who utilized their prosthetic limbs more frequently generally had a more positive clinical outcome and higher owner satisfaction whereas dogs with poor clinical outcomes and lower owner satisfaction spent less time in their prosthetic devices on average than dogs with positive outcomes and owner satisfaction. This finding is similar to reports in the literature describing parents' satisfaction and outcomes for pediatric patients with prosthetic limbs. Parents' ratings of satisfaction were correlated with the amount of time the prostheses were worn and the extent to which their children used their prostheses for activities in a variety of contexts (27). It is unknown at this time, however, if a prosthetic device is more likely to be utilized if a patient and owner are having a positive experience or if the positive experience is secondary to regular, consistent prosthesis use. In people, reasons reported for infrequent prosthesis use or prosthesis rejection were dissatisfaction with prosthetic comfort, function and control (28). On the other hand, prosthetic use appears to increase with functional ability which has been associated with experience and practice (29). It is probable that there are multiple factors which influence the relationship between prosthetic use, satisfaction, and outcomes.

It should be of note that both clinical outcome scores and owner satisfaction scores were higher in dogs who did not receive pre-habilitation. Additionally, application of post-prosthesis placement rehabilitation had no correlation or significant difference to owner satisfaction or clinical outcome scores. This would seem counterintuitive since rehabilitation/pre-habilitation should theoretically have a positive impact on outcome based on previous human-based literature which has shown a higher probability of return to mobility and autonomy with timely admission to a rehabilitation facility (30). It is likely, that this finding is due to unidentified confounding factors: for example the patients receiving more extensive care in the form of pre-habilitation may have been more severely clinically affected requiring more in depth intervention or did not respond well to the prosthetic due to clinical presentation. It would be expected that such patients may have lower clinical outcome scores and lower owner satisfaction scores. Again, a prospective study is needed to answer this question.

No correlations or significant differences were found between owner satisfaction scores and short- or long-term complications, however complication rates were generally high and should be of note when considering prosthetic limbs as a treatment option. Sores were by far the most common complication. Carr et al. previously reported patient factor complications such as sores and infections at 20.83% and Phillips et al. has reported a 58.3% complication rate (20, 21). This difference in reported complication rates may have to do with complication definitions, prosthesis manufacture, and variability in other patient factors, initial disease etiology, or the multi-center nature of the present study. All of these studies, including the present study, have demonstrated high rates of complications. Owners electing this treatment should be made aware of potential complications prior to electing a socket prosthesis as a treatment option.

The limitations of this study include the survey-based, owner-reported, retrospective nature. The response rate was relatively small considering previous indication by owners that they would not mind follow-up communication regarding their experience. It is possible that non-responders included dissatisfied owners. Multiple emails seeking survey participation were sent, though additional forms of communication, such as phone calls, were not pursued with non-responding individuals. The questionnaire used has not been previously validated and is based primarily on clinical experience and previously applied surveys; interpretation of data is likely influenced by researcher perception and experience. Survey interpretation by owners may have also been variable. It has been demonstrated that surveys contain an inherent bias due to the unknown factors of survey non-responders (31). Additionally, the data collected in this study were dependent on the owners' ability to recall events, outcomes, and feelings that occurred up to many years in the past. Medical records could not be obtained for all patients, therefore subjective owner responses could not be compared with imaging and veterinary records in more than half of the cases. The survey respondents were not selected in a random, rather from a database and a population of owners selecting a specific treatment option. These owners all had a significant time and often financial investment in the prescribed prostheses, therefore, response bias was highly likely. It may also account for the high level of owner satisfaction and discrepancies between owner satisfaction and clinical outcome. Owner perception can also be highly influenced by the care that they have received throughout treatment; while the prosthesis manufacturer was consistent for all owners surveyed, experience likely varied due to working with a multitude of veterinary practices prescribing and managing the devices. Experience level of the involved veterinary professional was not determined. Lastly, given the variability among patients evaluated in this survey, the sample size was too small to make definitive conclusions about prognostic factors that may influence prosthesis use.

Despite the aforementioned limitations, the overall high satisfaction rate and positive clinical outcomes demonstrated by this survey based study support continuation of further investigation into the use of socket prosthetic use in dogs. This study in combination with similar recent analysis of socket prostheses indicate that socket prostheses may be considered as an option for dogs with a partial limb due to amputation or congenital defects. Further clinical research using objective outcome measures is necessary to fully support the data represented in the present analysis.

## Ethics Statement

This study was carried out in accordance with the recommendations of Federal Policy for the Protection of Human Subjects, U.S. Department of Health and Human Services. Since the data gathered by the owners focused solely on their animals it does not meet the federal definition of a human subject and therefore did not require review by the Institutional Review Board.

## Author Contributions

TW, FD, and BS contributed to conception and design of the study. TW created the initial survey which was revised and edited by FD and BS. TW organized the data set and wrote the first draft of the manuscript. All authors contributed to manuscript revision, read and approved the submitted version.

### Conflict of Interest Statement

FD is a paid consultant of OrthoPets, LLC. The remaining authors declare that the research was conducted in the absence of any commercial or financial relationships that could be construed as a potential conflict of interest.

## Footnotes

^1^OrthoPets LLC, Denver, CO.

^2^SurveyMonkey, San Mateo, Cap.

^3^SAS Institute Inc., Cary, NC.
