# Supplementary material for: Retrospective Multi-Center Analysis of Canine Socket Prostheses for Partial Limbs
Source: Front Vet Sci. 2019 Apr 5;6:100. doi: 10.3389/fvets.2019.00100 (PMC6460115; doi:10.3389/fvets.2019.00100)
Supplement: Supplementary file 1 [file Data_Sheet_1.PDF]

Canine Prosthesis Survey

Dear dog owner,

We would like to ask for your help with our research. We are conducting a study with the goal of better understanding outcomes (eg: activity, function, owner satisfaction, etc.) associated with dogs who received a prosthesis following a partial-limb amputation or for a limb deformity. By gathering this information, we hope to improve the care and quality of life of dogs who will receive this treatment in the future.

We are contacting you because according to our records your dog was treated with a prosthesis provided by OrthoPets. We are asking for your participation in this short survey to answer some important questions that only you can answer. While it is possible to take this survey anonymously we would greatly appreciate if you would provide us at least your name and your pet's name so we can match the information gathered with your dog's medical records.

If your dog has passed away, please accept our most sincere condolences for your loss. We would still, however, genuinely appreciate your participation in our study, as this is an opportunity to help future patients. If this is the case for you and you wish to participate, please answer the questions to the best of your ability based on when your dog was still living.

Please be assured that all your information will be kept strictly confidential. No one outside our small research team will have access to your responses. Any aggregated data from this survey may be used for research purposes. We are not aware of any risks associated with taking this survey. If you have any questions or concerns regarding this study, do not hesitate to contact us.

Please note that the survey will take approximately 15-20 minutes and should be completed in one session so answers are not lost.

By selecting "continue with survey" below, you are giving us permission to include your information provided in this survey in our study. Additionally, you are giving us permission to access your dog's medical records for the purpose of this study. Any personal or identifying information provided will not be shared outside of our small study team.

a. Continue with survey
b. I do not wish to participate in the survey, but you may use my dog's records to provide additional information for your study
c. I do not wish to participate in this study

Personal identification and contact information:

1. Your surname
2. Your first name
3. Dog’s name
4. Preferred phone number

5. Preferred email address
6. Who was your dog's primary care veterinarian or the veterinarian who provided the majority of your dog's care during the time of prosthesis use?

7. Has your dog been seen at the Colorado State University Veterinary Teaching Hospital?
    a. Yes
    b. No

Questionnaire Section; All questions had a comment box available

1. My dog is...
    a. Alive
    b. Deceased

2. What breed is your dog?
    Open ended answer

3. What was your dog's age, or your best estimate of age, when your dog first received the prosthesis? (in years)
    a. Less than 1 yr
    b. 1
    c. 2
    d. 3
    e. 4
    f. 5
    g. 6
    h. 7
    i. 8
    j. 9
    k. 10
    l. 11
    m. 12
    n. 13
    o. 14
    p. 15
    q. 16
    r. Older than 16
    s. Don't know / don't remember

4. What was the reason for your dog's limb amputation?
    a. Trauma
    b. Cancer
    c. Congenital/birth defect requiring amputation or surgical revision
    d. I am not sure, I adopted my dog with a partial limb
    e. No amputation was performed, my dog was born with a partial limb
    f. Other (please specify)

5. For which limb was the prosthesis made?

a. Left front
b. Right front
c. Left hind
d. Right hind
e. Multiple limbs - please list which limbs

6. At what level on the limb was the amputation performed? For dogs who did not have an amputation, what is the level of the birth defect? Please refer to the above photo and corresponding numbers. You may use the "other" box if the amputation or defect was between the given numbers, if none of the options are applicable, or if you would like to provide additional description.

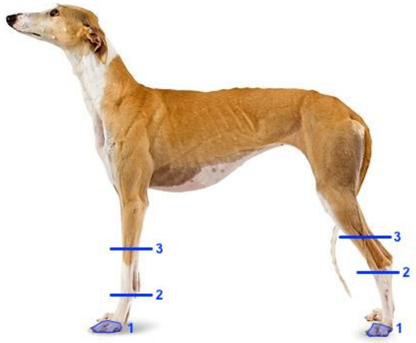

a. 1- Toe amputation(s)
b. 2- At the wrist/ankle or below the joint
c. 3- Mid-forearm (radius/ulna) or mid-"shin" (tibia/fibula)
d. Above line #3
e. I'm not sure (if you select this option, please also select the option that you think most closely represents your dog's limb)
f. Other (please describe); You may also use this box to explain if you answered "I'm not sure"

7. Approximately how much time passed between amputation and initial prosthesis placement?

a. Less than one month
b. Between one and two months
c. Between two and six months
d. Greater than six months
e. I'm not certain because I adopted my dog with a partial limb
f. Not applicable because my dog has a congenital defect and did not have an amputation.
g. Other (please specify)

8. How long has the prosthesis been used since obtaining it?

a. Less than six months
b. Six months to one year
c. One to two years

d. Two to three years
e. Three to five years
f. More than five years
g. Other (please specify)

9. How many days per week is the prosthesis worn on average?

a. 0
b. 1
c. 2
d. 3
e. 4
f. 5
g. 6
h. 7

10. Approximately how many hours per day does your dog wear the prosthesis (on days it is worn)?

a. 0
b. Less than 1
c. 1-3
d. 3-6
e. 6-9
f. 9-12
g. 12-15
h. 15-18
i. More than 18

11. For what purpose is the prosthesis used? (You may choose more than one answer)

a. Walks
b. Potty breaks
c. Play
d. Work (e.g. hunting, service work, sports, detection, moving livestock, etc.)
e. Mobility at home
f. All the time
g. Other (please specify)

12. How well does your dog walk with the prosthesis?

a. My dog uses the prosthesis almost every step
b. My dog uses the prosthesis most of the time, but holds the prosthetic limb up for some steps
c. My dog uses the prosthesis for some steps but most of the time he/she holds up the prosthetic limb
d. My dog never uses the prosthesis

13. How well has your dog adapted to using the prosthesis for other tasks? Please select specific tasks your dog does well with the prosthesis (you may choose more than one).

a. Running (places prosthesis and uses it as part of gait pattern)
b. Getting up from a laying position (using prosthesis for support)
c. Ascending or descending stairs (uses and occasionally leads with prosthesis)
d. Holding toys or bones with prosthesis
e. Digging with the prosthesis
f. My dog does not use the prosthesis well for any tasks
g. Other (please describe)

14. How easy is it to place the prosthesis (once you became well practiced)?
a. Very easy, no problems at all
b. Relatively easy
c. Acceptable, but not easy
d. Find it difficult but can usually manage
e. Very difficult, struggle with it most times

15. Does your dog like having the prosthesis placed?
a. Yes, my dog seems excited to have it placed
b. My dog doesn't seem to mind
c. No, my dog tries to avoid having it placed or runs away
d. Other (please specify)

16. What was the lifestyle/activity level of your dog prior to receiving the prosthesis? For dogs who had an amputation, please refer to your dog's lifestyle prior to limb loss (ie. before the problem started).
a. Sedentary, mostly inside during the day
b. Sedentary, mostly outside during the day
c. Mostly sedentary with periods of high energy
d. Moderately active
e. Very active
f. Working/athlete
g. Other (please specify)

17. How has your dog's activity changed since receiving the prosthesis? For dogs who had an amputation, please compare to your dog's lifestyle prior to limb loss (ie: before the problem started).
a. Moderate to marked increase in activity
b. Mild increase in activity
c. Same, no change
d. Mild decrease in activity
e. Moderate to marked decrease in activity
f. I don't remember

18. Has your dog experienced any short-term prosthesis complications? Examples: pressure or rub sores that resolved in less than 8 weeks and did not reoccur, quickly resolving pain associated with the amputation/defect site, swelling or size fluctuations of the amputated limb less than 8 weeks after amputation, etc.

a. No short-term complications
b. Sores
c. Pain
d. Swelling
e. Other (please specify)

19. Has your dog experienced any long-term prosthesis complications? Examples: Pressure or rub sores that lasted more than 8 weeks or reoccurred, chronic pain associated with the amputation/defect site, swelling or size fluctuations of the amputated limb more than 8 weeks after amputation, etc.
a. No long-term complications
b. Sores
c. Pain
d. Swelling
e. Other (please specify)

20. Did your dog perform rehabilitative exercises before receiving his/her prosthesis to improve later prosthesis use?
a. Yes
b. No

21. Did your dog perform rehabilitative exercises after receiving his/her prosthesis?
a. Yes
b. No

22. How often is/was physical therapy/rehabilitation performed after receiving the prosthesis (this includes at home exercises)?
a. Daily
b. Multiple times per week, but not daily
c. Once weekly
d. A few times per month
e. No rehabilitation was performed
f. Other (please specify)

23. How long was rehabilitation continued after receiving the prosthesis?
a. Less than one month
b. One to two months
c. Two to three months
d. Three to six months
e. Greater than six months, but eventually stopped
f. Life-long
g. No rehabilitation was performed

24. To the best of your knowledge, did your dog have any orthopedic disease when he/she received the prosthesis? Examples: torn anterior/cranial cruciate ligament (ACL), arthritis, hip dysplasia, loose kneecaps, fractures, etc.

a. No
b. Yes

25. To the best of your knowledge, did your dog develop any orthopedic disease after receiving the prosthesis?
a. No
b. Yes

26. To the best of your knowledge, did your dog have any neurologic disease when he/she received the prosthesis? Examples: Disc disease, degenerative myelopathy (DM), nerve damage in a limb, etc.
a. No
b. Yes

27. To the best of your knowledge, did your dog develop any neurologic disease after receiving the prosthesis?
a. No
b. Yes

28. How would you describe your level of satisfaction with your dog's prosthesis?
a. Very happy with outcome
b. Better than acceptable
c. Acceptable
d. Displeased
e. Very displeased

29. Based on your experience, would you choose a prosthesis for your dog again?
a. Yes
b. No

30. Knowing what you do now, how likely are you to recommend a prosthesis to another dog owner?
a. Very likely to recommend
b. Likely to recommend
c. Not recommend
d. Likely to discourage
e. Very likely to discourage

31. May we contact you for additional questions or follow-up to any of the questions on this survey?
a. Yes
b. No

32. This is a space for any additional comments or information you would like to provide regarding your dog's prosthesis (optional).
